# Supplementary material for: Promiscuous feeding on multiple adult honey bee hosts amplifies the vectorial capacity of Varroa destructor
Source: PLoS Pathog. 2023 Jan 19;19(1):e1011061. doi: 10.1371/journal.ppat.1011061 (PMC9851535; doi:10.1371/journal.ppat.1011061)
Supplement: S1 Tables — (DOCX) [file ppat.1011061.s001.docx]

**S1 Tables**

**Table A. Kruskal-Wallis ANOVA of DWV-A levels (log^10^ GE per bee) across groups**

| *Group* | *Unchallenged* | *Challenged* | *Challenged + DWV* | *Challenged + VDV1* |
| --- | --- | --- | --- | --- |
| *PreTrial Collection* | 0.96 | 0.027 | 0.40 | 0.48 |
| *Unchallenged* | - | <0.0001 | 0.004 | 0.025 |
| *Challenged* |  | - | 0.0004 | <0.0001 |
| *Challenged + DWV* |  |  | - | 0.61 |
| *Challenged + VDV1* |  |  |  | - |

**Table A**. Comparison of DWV-A levels between bees across all groups. There was a significant difference in DWV-A levels across groups (Kruskal-Wallis *H^2^* = 50.143, df=4, *P* < 0.0001). Dunn post hoc analysis with Benjamini-Hochberg method of DWV-A levels across groups. reported in the above table.

**Table B. Kruskal-Wallis ANOVA of VDV1 levels (log^10^ GE per bee) across groups**

| *Group* | *Unchallenged* | *Challenged* | *Challenged + DWV* | *Challenged + VDV1* |
| --- | --- | --- | --- | --- |
| *PreTrial Collection* | 0.09 | 0.19 | 0.2 | 0.36 |
| *Unchallenged* | - | 0.31 | 0.2 | < 0.0001 |
| *Challenged* |  | - | 0.73 | < 0.0001 |
| *Challenged + DWV* |  |  | - | <0 .0001 |

**Table B**. Comparison of VDV1 levels of bees across groups. There was a significant difference in VDV1 levels across groups (Kruskal-Wallis, *H^2^* = 91.044, df=4, P < 0.0001). Dunn post hoc analysis with Benjamini-Hochberg method of VDV1 levels across groups reported in the above table.

**Table C. Kruskal-Wallis ANOVA of DWV-A levels between non-parasitized bees across groups**

| *Group* | *Unchallenged* | *Challenged control* | *Challenged + DWV* | *Challenged + VDV1* |
| --- | --- | --- | --- | --- |
| *PreTrial Collection* | 0.99 | 0.045 | 0.51 | 0.45 |
| *Unchallenged* | - | < 0.0001 | 0.046 | 0.053 |
| *Challenged control* |  | - | 0.01 | 0.015 |
| *Challenged + DWV* |  |  | - | 1.00 |

**Table C**. Comparison of DWV-A viral levels between non-parasitized bees across all groups. (Kruskal-Wallis *H^2^* = 31.906, df=4, *p* < 0.0001) Dunn post hoc analysis with Benjamini-Hochberg method of DWV-A levels between non-parasitized bees across groups reported above.

**Table D. Kruskal-Wallis ANOVA of VDV1 levels between non-parasitized bees across groups**

| *Group* | *Unchallenged* | *Challenged control* | *Challenged + DWV* | *Challenged + VDV1* |
| --- | --- | --- | --- | --- |
| *PreTrial Collection* | 0.08 | 0.2 | 0.2 | 0.3 |
| *Unchallenged* | - | 0.23 | 0.33 | < 0.0001 |
| *Challenged control* |  |  | 0.82 | <0 .0001 |
| *Challenged + DWV* |  |  | - | < 0.0001 |

**Table D**. Comparison of VDV1 levels in non-parasitized bees across groups. VDV1 levels were significantly different for non-parasitized bees across all groups (Kruskal-Wallis *H^2^* = 71.774, df=4, *p* < 0.0001) Dunn post hoc analysis with Benjamini-Hochberg method of VDV1 levels between non-parasitized bees across groups reported above.

**Table E. Kruskal-Wallis ANOVA of DWV-A levels between parasitized bees across groups**

| *Group* | *Unchallenged* | *Challenged* | *Challenged + DWV* | *Challenged + VDV1* |
| --- | --- | --- | --- | --- |
| *PreTrial Collection* | 0.98 | 0.02 | 0.30 | 0.50 |
| *Unchallenged* | Na | < 0.0001 | 0.45 | 0.11 |
| *Challenged* |  | Na | 0.02 | 0.009 |
| *Challenged + DWV* |  |  | Na | 0.51 |

**Table E**. Comparison of DWV-A viral levels between parasitized bees. DWV-A levels of parasitized bees were significantly different across groups (Kruskal-Wallis *H^2^* = 35.852, df=4, *p* < 0.0001) Dunn post hoc analysis with Benjamini-Hochberg method of DWV levels between parasitized bees across groups reported above.

**Table F. Kruskal-Wallis ANOVA VDV1 levels between parasitized bees across groups**

| *Group* | *Unchallenged* | *Challenged* | *Challenged + DWV* | *Challenged + VDV1* |
| --- | --- | --- | --- | --- |
| *PreTrial Collection* | 0.06 | 0.09 | 0.21 | 0.69 |
| *Unchallenged* | Na | 0.76 | 0.19 | < .0001 *** |
| *Challenged* |  | Na | 0.46 | < .0001 *** |
| *Challenged + DWV* |  |  | Na | < .0001 *** |

**Table F**. Comparison of VDV1 levels in parasitized bees across groups. VDV1 levels were significantly different for parasitized bees across groups (Kruskal-Wallis *H^2^* = 32.683, df=4, *p* < 0.0001) Dunn post hoc analysis with Benjamini-Hochberg method of VDV1 levels between parasitized bees across groups reported above.
