# Supplementary material for: Promiscuous feeding on multiple adult honey bee hosts amplifies the vectorial capacity of Varroa destructor
Source: PLoS Pathog. 2023 Jan 19;19(1):e1011061. doi: 10.1371/journal.ppat.1011061 (PMC9851535; doi:10.1371/journal.ppat.1011061)
Supplement: Attachment — Submitted filename: ReviewerReplyDocumentPlosPathogens.docx [file ppat.1011061.s006.docx]

**Reply to Reviews Document**

| Dear Mr Lamas,    Thank you very much for submitting your manuscript "Promiscuous feeding on multiple adult honey bee hosts amplifies the vectorial capacity of Varroa destructor" for consideration at PLOS Pathogens. As with all papers reviewed by the journal, your manuscript was reviewed by members of the editorial board and by several independent reviewers. The reviewers appreciated the attention to an important topic. Based on the reviews, we are likely to accept this manuscript for publication, providing that you modify the manuscript according to the review recommendations.    This manuscript does two things very well: first, it uses the concept of vectorial capacity to describe transmission of a pathogen among honeybees by Varroa destructor; and second, in a series of experiments, it measures vectorial capacity. I found the paper to be engaging, interesting and new.  As someone who does not know a lot about this particular pathogen system, I thought an introductory paragraph about the system would perhaps be more effective than one that launches directly into a discussion of vectorial capacity.  I have made a few suggestions about that the discussion.  Please prepare and submit your revised manuscript within 30 days. If you anticipate any delay, please let us know the expected resubmission date by replying to this email.    When you are ready to resubmit, please upload the following:    [1] A letter containing a detailed list of your responses to all review comments, and a description of the changes you have made in the manuscript.    Please note while forming your response, if your article is accepted, you may have the opportunity to make the peer review history publicly available. The record will include editor decision letters (with reviews) and your responses to reviewer comments. If eligible, we will contact you to opt in or out    [2] Two versions of the revised manuscript: one with either highlights or tracked changes denoting where the text has been changed; the other a clean version (uploaded as the manuscript file).    Important additional instructions are given below your reviewer comments.    Thank you again for your submission to our journal. We hope that our editorial process has been constructive so far, and we welcome your feedback at any time. Please don't hesitate to contact us if you have any questions or comments.    Sincerely,    David L. Smith, Ph.D.  Guest Editor  PLOS Pathogens    Kirk Deitsch  Section Editor  PLOS Pathogens    Kasturi Haldar  Editor-in-Chief  PLOS Pathogens  [orcid.org/0000-0001-5065-158X](http://orcid.org/0000-0001-5065-158X)    Michael Malim  Editor-in-Chief  PLOS Pathogens  [orcid.org/0000-0002-7699-2064](http://orcid.org/0000-0002-7699-2064)    We would like to thank the editor and reviewers for the constructive feedback, and evident time placed into the review to make this a better paper for the readership audience. It was much appreciated by all co-authors. I would also like to apologize for not having originally included page numbers. Thank you for your patience with this, and they have been included in this next draft.  **Editors request for short introductory paragraph**  A short introduction was added. I attempted to introduce that Varroa have been introduced to Apis meliffera, that they are vectors, and feed on brood and adult bees. I chose to keep the introduction brief to maintain continuity and to maintain appeal to tick and mosquito vector biologists which we hope will be interested in the paper.   With respect to vectorial capacity:  **1. The formula for vectorial capacity in Garrett-Jones 1964 paper was extracted directly from Macdonald's 1952 paper, which introduced Ro into malaria epidemiology (and, indeed, into mathematical epidemiology). Since a lot of the work was done by Macdonald, I suggest also citing Macdonald (Ref: Macdonald, G. (1952). The analysis of equilibrium in malaria. Trop. Dis. Bull. 49, 813–829.)**  I have updated the introductory line to include the citation recommended. For simplicity I left the text as is.   2. **I think it's more accurate to say that it has been applied to "human-mosquito-pathogen" systems. Vectorial capacity predicts the number of infectious bites by vectors (not vectors) that would eventually arise from all the vectors blood feeding on a single infectious human on a day**.  I made the corrections suggested directly into the text.   **3. In the opening paragraph, there is a reference to a figure but no number was given.**  I deleted the reference to the figure. Thank you for catching this. Originally we had included a figure depicting the VC equation, but had removed it due to our already high figure counts.  4. **The set of refs 3-5 at the end of the first paragraph is a bit eclectic. I suggest (with some shame) citing a systematic review. Full disclosure, I was an author on this systematic review. (The reference is: Reiner RC, et al. (2013). A systematic review of mathematical models of mosquito-borne pathogen transmission: 1970-2010. J. R. Soc. Interface 10, 20120921.)**  I replaced the references at the end of the first paragraph with the suggested systematic review. Thank you for the authorship disclosure; this citation cleans up the end of the first paragraph nicely.  **5. The discussion that follows about sensitivity to parameters seemed a bit confusing to me. In discussing "this one parameter of VC models" that has a disproportionate influence on transmission, the authors cite a paper that emphasizes mosquito mortality / lifespan. I think the authors are more interested in the feeding rate. The history of this idea is a bit involved, so I will elaborate: Macdonald's other 1952 paper made the cases that mosquito longevity was critically important for transmission (Macdonald, G. (1952). The analysis of the sporozoite rate. Trop. Dis. Bull. 49, 569–586.). The paper launched new research, and Macdonald revisited this idea in a paper published in 1955 about eradication. Measuring longevity has become a sort of holy grail in studies of pathogen transmission by mosquitoes. In this vector-transmitted pathogen system that the authors are describing, where the main mode of transmission is mechanical (if I understand it correctly), the factor that Macdonald was discussing (surviving the parasite's EIP) is not relevant. In this system, the authors are emphasizing that the feeding rate (in particular, host switching) does, indeed, affect transmission in at least two ways. (In mosquitoes, since blood is provisioned into eggs, which are laid and hatch into adults, the blood feeding rate could be assigned a third effect, at least for purposes of weighing vector control. I'm not sure if it applies to this system.). The authors would not be the first to draw attention to the blood feeding rate, but IMO, the paper by Novoseltsev et al. is chasing the argument about lifespan. It was Garrett-Jones, not Macdonald, who drew a bit more attention to the human blood feeding rate, especially when looked at in the context of his other paper from 1964 on the human blood index. I would suggest the authors emphasize that this paper focuses on the feeding rate and host switching (in this case), and not one that focuses on vector mortality. Not many papers on pathogen transmission by vectors have put such an emphasis on the feeding rate, which increases the novelty.**  I removed the citation by Novoseltsev et al. as suggested and replaced it with Garret-Jones 1964 paper, as well as (Smith et al. 2007) PlosBiology paper. The first sentence of the next paragraph was merged into a new last sentence of this paragraph to keep continuity and to take the suggestion that we should emphasize we are investigating the feeding rate.  Reviewer Comments (if any, and for reference):  Reviewer's Responses to Questions  **Part I - Summary** Please use this section to discuss strengths/weaknesses of study, novelty/significance, general execution and scholarship.  **Reviewer #1: Overall, I enjoyed this paper and would hope to see it published. I think the mix of vector-transmission and between-host transmission of important bee viruses is a fascinating topic. The result of parasitized bees which died quickly after Varroa feeding may be conferring protection to non-parasitized nestmates by limiting opportunities for host to host transmission, whereas long lived survivors may elevate risk to nestmates, is particularly interesting. With my modeller hat on, and associated biases, I would encourage these authors to engage with vector-borne disease modellers. There is already scope for some basic first-principles analyses using a Ross-Macdonald framework; and, the interaction might even foster ideas for new experiments.**  Thank you very much for this comment. The lines you highlighted is a finding we believe has general relevalance in many infectious disease systems and will be important as we develop models for Varroa-DWV-honeybee.  Reviewer #2: The study provides new insight on Varroa host-switching behavior on adult bees. The study is of importance and interest. The study provides interesting results on virus level in bees that relate to parasitism by Varroa on the host or not. I think that the paper can benefit from improved introduction and the same references can be used in discussion.  **Part II – Major Issues: Key Experiments Required for Acceptance** Please use this section to detail the key new experiments or modifications of existing experiments that should be absolutely required to validate study conclusions.  Generally, there should be no more than 3 such required experiments or major modifications for a "Major Revision" recommendation. If more than 3 experiments are necessary to validate the study conclusions, then you are encouraged to recommend "Reject".  Reviewer #1: (No Response)  Reviewer #2: The experiments were well performed.  **Part III – Minor Issues: Editorial and Data Presentation Modifications** Please use this section for editorial suggestions as well as relatively minor modifications of existing data that would enhance clarity.  Reviewer #1: **Mostly, the readability of the results needs improving. 5 multi-part figures and 12 tables is inundating. The authors have done a huge amount of work and I applaud them for** **packing it together into a single paper rather than 3 smaller papers, but some effort is needed to make it more manageable. Please see below some more specific comments.**  Thank you. We had considered breaking this apart into smaller papers, and instead took the time to write a cohesive story in this manuscript. We have addressed the specific examples mentioned below and we hope in a way that enhanced clarity.  **Figure 1.3 shows the number of bees parasitized by individual Varroa over time. It distinguishes Varroa which died from those that survived the 15d trial by colour. The authors accounted for longevity of the Varroa by dividing the number of switches a Varroa would make by the number of days that Varroa survived in the experiment. I think it is this that is displayed in Fig1.3 (because some data points have non-integer values on the y-axis and a mite cannot parasitize 0.3 of a bee). But I don’t know how some Varroa on day 15 had parasitized 8 bees (i.e., 120 bees parasitized divided by the 15 days survived). I think I've misinterpreted this figure and associated text. Some more explicit description of exactly what is plotted in Fig1.3 would be appreciated.**  I added two lines to the caption to give clarity to the figure. I replaced the figure with an updated figure. I went into R and used the jitter function to offset points to prevent overlapping. However, this time I applied it only to the x-axis. Now all points on the y-axis fall on a whole number as they should.  I originally described two different analysis in the same paragraph. I had provided analysis on the number of switches and analysis on the number of parasitized hosts, which was confusing. I edited this for clarity by moving some lines into a second paragraph. I kept descriptions on the host switching rate and who contributed to parasitized hosts. I believed this distinction is important because mites can revisit a previously fed upon host. As a result, it is possible for mites to have more switches than different hosts they fed upon.  **Table 1.3 and associated text: after reading through this 3 times I gave up. I sympathise that it is a complicated set of comparisons being made between parasitised and non-parasitised bees** **within the same treatments versus non-parasitised bees between different treatments etc., but, I think the ms would benefit from the authors coming up with a better way of encapsulating exactly which comparisons yielded which result. Would there be a pictorial way of depicting the comparisons and combining the results from multiple tables (lessening the tirade of tables)?**  I edited the table for clarity. I removed two columns which we believed leaving would be universally confusing to readership and not benefit the data. I placed the information that was removed directly into the text (lines 438-439). I gave more detailed descriptions in the caption for the remaining data, and referenced the particular column.  **Table 1.4: the final column header is ‘Mean percentage of non-parasitized to parasitized nestmates’ but the table legend states details are provided for ‘the average ratio of parasitized to non-parasitized bees in each group’. One of these two depictions is wrong. Why not present raw numbers with prevalence of parasitism in brackets afterwards?**  You are correct that the header and the depiction do not match. The final column description was updated, and a full description was provided for clarity and the header was updated for clarity.  **Reviewer #2: Abstract – what does mean „now endemic in honeybee populations” – it appears misleading**  The line was updated for accuracy with “endemic in honey bee populations in Varroa-infested regions. DWV was present in bee populations prior to the spread of Varroa destructor. Prior to Varroa expansion it was possible to find populations of DWV free colonies. Several studies have shown immense amplification of the pathogen once Varroa was established in a new region.  ”. **Importantly, DWV can be identified in other bee species – thus, you should provide exact information**  We concluded after discussion that DWV infection in non-Apis m. bee species was outside of the scope and relevance of this paper. DWV and BQCV spillover from managed honeybees to native bees is a major concern and is garnering global attention with the explosion of backyard beekeeping and the impacts high density commercial management of honeybees for pollination has on native bees. However, we believe our paper would most benefit a readership audience by focusing on the gap in knowledge our paper is addressing: feeding on adult bees by Varroa is understudied and there is a wealth in knowledge in vectorial capacity in mosquitoes that has not been applied to Varroa-DWV-honeybee. We concluded that DWV replication in other bees and spillover in other bees would detract from the continuity in the paper without improving information delivery to the readership. Additionally, this information is easily accessible to the PLOS readership as there have been recent reviews and prominent papers that have garnered much attention and provide this exact information.  **Introduction It can be recommended: the manuscript could benefit from including information about pathways that Varroa affects. To some extent, there are similarities to ticks. Ticks are mites and thus are more related to Varroa than mosquitoes. The pathways can also be similar.**  Respectfully I was unsure what the review meant by this comment. After review by co-authors we decided not to add information specific to ticks, in hopes to keep continuity and the message on point with host switching rates and vectorial capacity.  **It is important to introduce, that the mite affects bees. In addition, the mite can transmit also other viruses, not only deformed wing virus. For instance, acute paralysis virus it is of high importance.**  There are other viruses linked to bee disease but none are so tied to vector-host dynamics as DWV and its variants. Respectfully we decided to keep the focus in this paper on DWV and VDV1 vectored by Varroa as there are recent virome studies and reviews on other viruses vectored or transmitted in honeybee colonies.  **Further, the mite strongly affected virome of honey bees – immortally, the virome of Varroa naïve bees (Varroa was until now absent in Australia) is different than in other countries. Thus, information related to this can be recommended to introduce. At page 4 “with associated viruses” – it is superficial information without any reference. Authors should rather provide information which viruses Varroa transmits, which viruses were detected in the mite to be potentially transmitted to bees. In addition, virome of honey bees is of importance, because the mite then transmits viruses related to the particular colonies / sites.**  **Page 4 “North America” – why only North America? – the mite shifted to European honey bees in Europe much earlier and then it spread worldwide – most recently to Australia.\**  “North America” was deleted and instead replaced with globally.  **Page 4 “a pathogen efficiently vectored by the mite” it is question if the virus is “only” transmitted by the mite or if it replicates in the mite. Study that supports “only” transmission can be provided.**  This is an important consideration in DWV-A and VDV1 transmission by mites, and hotly debated at the moment. We address this on page 30 (lines 622 – 624) in the discussion and give two prominently cited papers for this topic. For continuity and conciseness within the introduction we elected to leave the manuscript unchanged in this regard.  **Page 5 “Varroa feeding and development on immobile honey bee brood (larvae and pupae),” – the development of bees and pathways is affected and some reference is missing.**  The references Posada-Florez et al 2019, Kanbar 2003, and Yang 2007 were added to provide background on the statement about feeding on developing pupae. No citation was provided at the end of that sentence in regards to the little being known about feeding patterns on adult bees, since immediately following in the next paragraph a full description of what is known with references are provided.  **Further, you should consider provide studies that detected DWV in other species that do not host Varroa.**  Respectfully we replied to a similar comment previously.  **Page 5 – “let alone quantitative estimates of the crucial host-shifting rate, as needed for vector capacity (VC) models of transmission and disease” there were analyzed adult mites – references can be provided. The viruses were identified using PCR, but some viruses were also identified in Varroa at protein level using proteomics.**  I added garret-Jones reference to the sentence mentioned here since that paper brings attention to the biting rate. Our manuscript references all known published papers which have described host switching on adult bees earlier in this paragraph on page 5. We did not repeat using those references at the final sentence mentioned in this comment due to redundancy. “The viruses were identified using PCR, but some viruses were also identified in Varroa at protein level using proteomics.” Thank you for noticing that we neglected to reference our methods for viral detection in our methods. This was an oversight on my part. I have included a description with citations to exact methods in the methods section to address this comment. Shortly: We used real time quantitative RTqPCR after RNA extraction and conversion of RNA to cDNA to detect viruses in this study. (page 14, lines 285 – 291)   **Page 6 – “on the viral state of previous hosts.” – virome studies of honey bees can be used**  Respectfully, I am not sure what is being asked here. After returning to the manuscript we do not believe a citation is needed. We are giving a prelude at the end of the introduction informing the readership broadly what our findings were, and framing the scope of the future discussion.   **You used incola of DWV-A and VDV1 – which viruses (DWV strains) predominate in Varroa parasitized bees? – adult bees? Virome studies can be used.**  DWV-A is a predominant strain of DWV, though VDV1 is potentially going to take this role. We used DWV-A inoculum and VDV1 inoculum, both clone derived and with published papers which describe the inoculums. The inoculums were previously shown to cause infection in those studies. We already had the citation for the DWV inoculum. I am adding the ref. (Posada, 2020) for the VDV1  **It appears that bees die earlier if bees are challenged to Varroa and DWV or VDV1 – but Varroa transmits also viruses – which viruses were in the mite?**  I am not entirely sure what is being asked here but I think an important question is being asked. In general Varroa can transmit many viruses. This is well reviewed in Beaurepaire et al, 2020. This reference has been added to the manuscript. Specific to our study, and how the experimental design was created, other viruses in our mites was an unavoidable possibility that was minimized but also an unlikely confounder. Previous research has shown Varroa can harbor viruses (or virus strains) without transmitting them to hosts. Posada et al, 2019 and Moore et al 2011 reported such results. Further, as a largely mechanical vector (albeit VDV1 which some evidence shows can replicate inside of mites) it is highly unlikely a virus harbored inside the vector at very low levels would be transmitted to a host through parasitism. Our pre-trial experimental design specifically addressed this in several ways. First to reduce variability in both hosts and vectors in our work, every study used single colony sourced mites, and single colony sourced emerged bees. Mites were sourced from a single colony which although was infested, did not exhibit signs of disease. Second, mites were all treated the same before being randomized into experimental groups, which included placing the mites on pupae to feed prior to the start of the trial. Pupae were collected from an ostensibly healthy colony, with a zero detection of mites in an alcohol wash and without showing any clinical signs of dease. This allowed all mites, whether they were in non-reproductive state for various times(previously called phoretic) to be equalized prior to the trial. We also acknowledge this sentiment, if that is what is being asked, clearly in our mansuscript in the methods section. On page 13-14, lines 276 to 282 we define what was considered a control mite, an infectious mite, and then acknowledge that heterogeneity in the viral loads in mites could not be accounted for. We did not report results or make claims in our manuscript that would warrant sampling individual mites used in this work.   **Could you provide more information/discussion about presence of viral strains in bees?**  The bees collected for the experiment (Fig 1.5) were free of either DWV or VDV1. No high levels of DWV or VDV1 developed in the bees which were not exposed to Varroa mites.  **Some studies are available. It is of importance that controlling of Varroa infestation can affect occurrence of DWV strains only to some extent.**  We did not provide a comprehensive description of viral types or strains in honeybee populations in our region or globally. In the introduction we cite several papers when we reference viruses as culprits to colony loss (Steinhauer 2018 and Carreck 2009) which mention other viruses suspected to impact colony health. We did choose to direct the readership to Wilfert et al 2016 early in the introduction because of their comprehensive analysis of DWV and its global spread. Our manuscript in laboratory trials saw amplification of DWV-A with the presence of mites, and no amplification in unchallenged groups without mites which has been a finding with Wilfert et al, and prominently Martin, 2019 in the Hawaiian archipelago.   **risk of naïve Varroa - ? not clear, could you please explain – does it mean Varroa without virus? Or bees naïve to Varroa**  A naive host and a naïve vector are non-infectious. This is terminology is commonly used when describing non-infectious mosquitoes and we are adopting the language here. “Naïve varroa” means Varroa that are not infectious, i.e. do not harbor high loads of a pathogen. Specifically in this part of the discussion 691 – 695 the manuscript brings attention to the fact that Varroa may be naïve, in a non-infectious state, and acquire virus and become infectious from viral circulation between host to host. The concept that “mites can be at risk” is epidemiologically sound even though it appears to be a misnomer because we so often only think of hosts as being at risk. However, risk is simply a mathematical probability of an event occurring. So in complex disease systems such as Varroa-DWV-Bee or as we mention here WNV and Tembusu virus, which all have multiple efficient host-host and vector-host routes, the vector itself would have increased risk under certain conditions.   Minor corrections: correct “population.-The proportion”  PLOS authors have the option to publish the peer review history of their article ([what does this mean?](https://nam11.safelinks.protection.outlook.com/?url=https%3A%2F%2Fjournals.plos.org%2Fplospathogens%2Fs%2Feditorial-and-peer-review-process%23loc-peer-review-history&data=05%7C01%7Cdsonensh%40odu.edu%7C4dd137577ec9417e568408da88619001%7C48bf86e811a24b8a8cb368d8be2227f3%7C0%7C0%7C637972249850025892%7CUnknown%7CTWFpbGZsb3d8eyJWIjoiMC4wLjAwMDAiLCJQIjoiV2luMzIiLCJBTiI6Ik1haWwiLCJXVCI6Mn0%3D%7C3000%7C%7C%7C&sdata=hcqg1m3zkMwssod3TlxhESOjyYQmpas5rZ33ujDixlk%3D&reserved=0)). If published, this will include your full peer review and any attached files.   If you choose “no”, your identity will remain anonymous but your review may still be made public.   **Do you want your identity to be public for this peer review?** For information about this choice, including consent withdrawal, please see our [Privacy Policy](https://nam11.safelinks.protection.outlook.com/?url=https%3A%2F%2Fwww.plos.org%2Fprivacy-policy&data=05%7C01%7Cdsonensh%40odu.edu%7C4dd137577ec9417e568408da88619001%7C48bf86e811a24b8a8cb368d8be2227f3%7C0%7C0%7C637972249850025892%7CUnknown%7CTWFpbGZsb3d8eyJWIjoiMC4wLjAwMDAiLCJQIjoiV2luMzIiLCJBTiI6Ik1haWwiLCJXVCI6Mn0%3D%7C3000%7C%7C%7C&sdata=52JY%2FkoTvql%2BhlLSKZNoR12NYP1ifSnVEo623zMmKcE%3D&reserved=0).  Reviewer #1: **Yes:** Laith Yakob  Reviewer #2: No        Figure Files:    While revising your submission, please upload your figure files to the Preflight Analysis and Conversion Engine (PACE) digital diagnostic tool, <https://pacev2.apexcovantage.com>. PACE helps ensure that figures meet PLOS requirements. To use PACE, you must first register as a user. Then, login and navigate to the UPLOAD tab, where you will find detailed instructions on how to use the tool. If you encounter any issues or have any questions when using PACE, please email us at [figures@plos.org](mailto:figures@plos.org).    Data Requirements:    Please note that, as a condition of publication, PLOS' data policy requires that you make available all data used to draw the conclusions outlined in your manuscript. Data must be deposited in an appropriate repository, included within the body of the manuscript, or uploaded as supporting information. This includes all numerical values that were used to generate graphs, histograms etc.. For an example see here: [http://www.plosbiology.org/article/info%3Adoi%2F10.1371%2Fjournal.pbio.1001908#s5](https://nam11.safelinks.protection.outlook.com/?url=http%3A%2F%2Fwww.plosbiology.org%2Farticle%2Finfo%253Adoi%252F10.1371%252Fjournal.pbio.1001908%23s5&data=05%7C01%7Cdsonensh%40odu.edu%7C4dd137577ec9417e568408da88619001%7C48bf86e811a24b8a8cb368d8be2227f3%7C0%7C0%7C637972249850025892%7CUnknown%7CTWFpbGZsb3d8eyJWIjoiMC4wLjAwMDAiLCJQIjoiV2luMzIiLCJBTiI6Ik1haWwiLCJXVCI6Mn0%3D%7C3000%7C%7C%7C&sdata=cXrS%2B8MmpIuev6knO96Em7OZBSnM2I1DH%2B61aS1CSBw%3D&reserved=0).  Reproducibility:  To enhance the reproducibility of your results, we recommend that you deposit your laboratory protocols in [protocols.io](http://protocols.io), where a protocol can be assigned its own identifier (DOI) such that it can be cited independently in the future. Additionally, PLOS ONE offers an option to publish peer-reviewed clinical study protocols. Read more information on sharing protocols at [https://plos.org/protocols?utm_medium=editorial-email&utm_source=authorletters&utm_campaign=protocols](https://nam11.safelinks.protection.outlook.com/?url=https%3A%2F%2Fplos.org%2Fprotocols%3Futm_medium%3Deditorial-email%26utm_source%3Dauthorletters%26utm_campaign%3Dprotocols&data=05%7C01%7Cdsonensh%40odu.edu%7C4dd137577ec9417e568408da88619001%7C48bf86e811a24b8a8cb368d8be2227f3%7C0%7C0%7C637972249850025892%7CUnknown%7CTWFpbGZsb3d8eyJWIjoiMC4wLjAwMDAiLCJQIjoiV2luMzIiLCJBTiI6Ik1haWwiLCJXVCI6Mn0%3D%7C3000%7C%7C%7C&sdata=rSM1Qq%2BQjFN4VzrfQrmd1C1nCMHeYNWnO4dJbPo634Y%3D&reserved=0)    References:  Please review your reference list to ensure that it is complete and correct. If you have cited papers that have been retracted, please include the rationale for doing so in the manuscript text, or remove these references and replace them with relevant current references. Any changes to the reference list should be mentioned in the rebuttal letter that accompanies your revised manuscript. If you need to cite a retracted article, indicate the article’s retracted status in the References list and also include a citation and full reference for the retraction notice. | | |
| --- | --- | --- |
