# Supplementary material for: Promiscuous feeding on multiple adult honey bee hosts amplifies the vectorial capacity of Varroa destructor
Source: PLoS Pathog. 2023 Jan 19;19(1):e1011061. doi: 10.1371/journal.ppat.1011061 (PMC9851535; doi:10.1371/journal.ppat.1011061)
Supplement: Attachment — Submitted filename: AnnotadedChangesForReviewers.docx [file ppat.1011061.s007.docx]

**Response to Reviewer Comments**

Reviewer comments: **The authors are encouraged to revise their manuscript one more time, paying close attention to the descriptions in the text of every quantity that is discussed. In particular, the first time any quantitative term is introduced in text, the authors should take great care to describe each term in detail, so that when the shorter phrase is used later, there is no doubt about what the quantity describes. In particular, we invite the authors to modify how they describe the term relative risk.**

The following are our responses to reviewer comments imbedded in our last email from PlosPathogens.

236-241, Switch and switching rate are defined lines

241-243 within the methods section we define what the host switching rate is and how it was calculated

291 – 327 ,In methods, under Statistical analysis lines definitions for relative risk, survivorship, and parasitized hosts.

294 we defined the per day switching rate of a mite

265 – 267 within the methods section we removed a redundant line. By doing so we believe it increases readability.

319 – 324 within the methods section we added specific detail describing how relative risk was calculated, and defined the two comparisons that were made. We then made sure the terminology used first here in the methods section is the same terminology used later in the results section.

336 – 337 within the results section we changed the title of the first header to more appropriately describe the results immediately following.

356- 383 To reduce confusion between switching rate and the number of parasitized hosts a mite contributes, we moved lines about switching rates so they are all now together, and then provided a new paragraph when referring to the number of parasitized hosts.

372 – 379 within the results section we changed the layout of the paragraph. Now the beginning of the section entirely reports on host switching and the host switching rate. We needed to report data which is slightly different, how high and low frequency switching mites contribute to the number of parasitized hosts, without confusing the reader. We did this by immediately following with table 2 and the header **Mite switches and contribution to parasitized bees.** The following text then transitions into contribution to parasitized hosts. We believe this will improve readability and reduce confusion for the readership.

372 We changed the header to more accurately describe the following paragraph

435 we deleted an unneeded sentence so that results are reported with better flow

446 -452 Prior to reporting our risk and relative risk data, we chose to define these terms, and which groups were being compared. This is repetitive since it is stated in methods, but after consideration we decided this would be best for the readership. This is a long document that could have been divided into multiple smaller papers. At this later point in the document clearly knowing the groups will aid readership, and we believe it did not tarnish the readability of the section.

**Table 3** We changed the header in column 4

459 We changed the header to more accurately describe the following paragraph

476 -477 We added a single introductory line so readership knows we are highlighting length of exposure of non-parasitized bees to parasitized bees.
